# Integrative multimodal framework combining undercarboxylated osteocalcin and medial prefrontal cortex morphometry for prediction of cognitive decline in osteoporosis: a machine learning study

**DOI:** 10.3389/fneur.2026.1950788

**Published:** 2026-09-16

**Authors:** Xiaomeng Tong, Yun Zhou, Yongtong Cao, Chengwu Han

**Affiliations:** Department of Laboratory Medicine, China-Japan Friendship Hospital, Beijing, China

**Keywords:** brain-bone axis, cognitive decline, grey matter volume, machine learning, neuroimaging biomarkers, osteoporosis, undercarboxylated osteocalcin

## Abstract

**Objective:**

This study investigates the mechanistic link between age-related osteoporosis (OP) and cognitive decline, with a particular emphasis on the neuroprotective role of undercarboxylated osteocalcin (ucOC)—the biologically active form of osteocalcin—and its influence on brain structural integrity and cognitive performance. We further explored the translational potential of integrating neuroimaging features, and machine learning algorithms to develop predictive models for early identification of cognitive deterioration in OP patients.

**Methods:**

We conducted a comprehensive voxel-based morphometry (VBM) analysis across two independent datasets. Voxel-wise comparisons between OP patients and healthy controls were performed to characterize grey matter volume (GMV) alterations. Correlation and mediation analyses were subsequently employed to elucidate the relationships among serum ucOC levels, GMV, and cognitive function. Brain regions exhibiting significant mediation effects were identified as ucOC-associated neuroimaging biomarkers in Dataset 1, and their GMV values were used as fixed features to develop machine-learning models for predicting 1-year cognitive decline in OP patients in the independent Dataset 2, evaluated through a leakage-free leave-one-out cross-validation procedure.

**Results:**

OP patients exhibited significantly lower Montreal Cognitive Assessment (MoCA) scores and reduced GMV in the medial prefrontal cortex (mPFC) relative to healthy controls. Notably, serum ucOC, but not carboxylated osteocalcin (cOC), demonstrated significant positive correlations with both MoCA scores and mPFC GMV in OP patients. Mediation analysis further confirmed that mPFC GMV statistically mediates the relationship between ucOC and cognitive function. Machine-learning models integrating mPFC GMV achieved good discriminative performance in leakage-free validation in Dataset 2 (XGBoost: AUC = 0.84, 95% CI [0.79, 0.88]), classifying OP patients at elevated risk of cognitive decline over a 1-year follow-up period.

**Conclusion:**

Our study establishes undercarboxylated osteocalcin (ucOC) as a mechanistically relevant, circulating biomarker of cognitive resilience in osteoporosis, and identifies medial prefrontal cortex grey matter volume (mPFC GMV) as its structural neuroimaging correlate for ucOC. Importantly, XGBoost yields a promising predictive framework that achieved good discriminative accuracy for 1-year cognitive decline risk.

## Introduction

Age-related osteoporosis (OP), characterized by progressive decline in bone mineral density and deterioration of bone microarchitecture, represents a chronic skeletal disorder that substantially increases fracture susceptibility and imposes a considerable global healthcare burden (1, 2). As populations worldwide continue to age, the prevalence of OP is escalating, amplifying its socioeconomic impact (3). Increasingly, OP is recognized not merely as a localized bone disorder but as a systemic condition with far-reaching implications for brain health. Epidemiological evidence has consistently linked OP to neurodegenerative diseases, including dementia and Alzheimer’s disease (AD), suggesting shared pathophysiological underpinnings between skeletal and cognitive deterioration (4–7). For instance, individuals diagnosed with AD frequently exhibit reduced bone mineral density and elevated fracture risk, reinforcing the notion that bone health and cognitive function are intimately intertwined (8–10).

The co-occurrence of OP and neurodegenerative disorders is not coincidental but rather reflects shared pathological mechanisms, including chronic low-grade inflammation, oxidative stress, estrogen deficiency, and altered angiogenesis (7, 11). The emerging concept of the brain-bone axis has provided a compelling mechanistic framework for understanding these bidirectional interactions, revealing that the central nervous system and bone metabolism engage in complex endocrine crosstalk (12–15). Multiple bone-derived secretory proteins, including osteocalcin (16–18), fibroblast growth factor 23 (FGF23) (14, 16, 17), and sclerostin (18), have been demonstrated to cross the blood–brain barrier and modulate neuronal activity, neurotransmitter synthesis, and synaptic plasticity. Among these, osteocalcin—particularly its undercarboxylated form (ucOC)—has garnered particular attention as a multifunctional hormone with potent neuroprotective properties. Unlike its carboxylated counterpart (cOC), which is primarily sequestered within the bone matrix, ucOC is actively released into circulation during bone resorption and represents the biologically active form capable of crossing the blood–brain barrier (19). Once in the brain, ucOC accumulates in critical regions including the brainstem, thalamus, and hypothalamus, where it binds to neuronal Gpr158 receptors and modulates the synthesis and signaling of serotonin, dopamine, and GABA. These findings position ucOC as a unique bone-derived endocrine signal with direct neuromodulatory capacity, offering a mechanistic basis for the observed comorbidity between OP and cognitive impairment.

Given the rising prevalence of both OP and cognitive decline, there is an urgent need for effective strategies to monitor, predict, and intervene against cognitive deterioration, particularly in patients with compromised bone health (1, 20). The identification of reliable biomarkers that can detect early signs of neurodegenerative change is essential for developing proactive diagnostic tools (2, 4). In this regard, neuroimaging—particularly structural magnetic resonance imaging (MRI)—offers an unparalleled non-invasive window into brain structure (21). MRI-based voxel-based morphometry (VBM) enables sensitive, whole-brain quantification of grey matter volume (GMV) alterations, providing objective neuroimaging biomarkers that may correlate with circulating bone-derived factors and cognitive status (22, 23). By linking peripheral serum biomarkers such as ucOC to region-specific structural brain changes, we can establish a multimodal diagnostic framework that captures both systemic and central nervous system dimensions of disease.

Building upon the complementary strengths of serum biomarker quantification and neuroimaging, the integration of machine learning algorithms offers a transformative approach to predictive modeling in neurodegenerative disease (24). Machine learning excels at identifying complex, non-linear patterns and latent relationships within high-dimensional multimodal datasets, surpassing the predictive capacity of traditional statistical methods (25). When applied to neuroimaging data, machine learning can capture subtle anatomical signatures of early cognitive impairment that may elude conventional analysis (26, 27). Crucially, the synergistic combination of serum biomarkers (e.g., ucOC), structural neuroimaging features (e.g., mPFC GMV), and machine learning algorithms enables the construction of robust, individualized predictive models. Such multimodal integration addresses the inherent heterogeneity of patient populations and leverages complementary information across biological scales, from molecular circulation to macroscopic brain structure, ultimately facilitating earlier detection, more accurate risk stratification, and tailored therapeutic strategies.

In the present investigation, we pursued three integrated objectives. First, we sought to identify specific brain regions where GMV is associated with serum levels of bone-secreted proteins—particularly the biologically active ucOC—in individuals with OP. Second, we aimed to dissect the mechanistic relationships among ucOC, regional GMV, and cognitive function through correlation and mediation analyses. Third, we identified ucOC-associated neuroimaging biomarkers in Dataset 1 and developed machine learning-based predictive models using these fixed imaging markers to predict cognitive decline over a 1-year follow-up period in the independent Dataset 2, evaluated with a leakage-free cross-validation procedure; neuroimaging findings were independently replicated across both cohorts. This multimodal, translational approach is designed to advance our understanding of the bone-brain axis and to establish an evidence-based framework for early, personalized prediction of cognitive decline in OP patients.

## Materials and methods

### Patient population and clinical assessment

The present study received approval from the ethics review committee of China-Japan Friendship Hospital, and informed written consent was obtained from each participant. We collected two datasets for this study. Dataset 1 included sixty-three right-handed patients with osteoporosis (OP) and sixty right-handed healthy controls, matched for age and education. Dataset 2 was collected in the same center and consisted of thirty-two right-handed patients with OP and thirty-one right-handed healthy controls, also matched for education. In both datasets, cognitive performance was assessed for patient cohorts and healthy controls during the initial data collection phase and then again after a 1-year follow-up using The Montreal Cognitive Assessment (MoCA). Additionally, for participants in dataset 1, serum undercarboxylated osteocalcin (ucOC) and carboxylated osteocalcin (cOC) levels were measured using the ELISA method (Details See Supplementary Methods).

### MRI data acquisition and voxel-based morphometry (VBM)

Structural MRI data were processed using the Computational Anatomy Toolbox (CAT) within the Statistical Parametric Mapping software suite (SPM12, version 6906; Wellcome Trust Centre for Neuroimaging), which was interfaced through MATLAB 2023a (MathWorks Inc., Natick, Massachusetts, USA). Voxel-Based Morphometry (VBM) analysis was performed to investigate the brain structural changes in OP patients (Details See Supplementary Methods).

### Statistical analysis

To investigate differences in clinical metrics between OP patients and healthy controls, we conducted two-sample *T*-tests to explore the differences in ucOC, cOC at the baseline, and MoCA scores at both the baseline and after a 1-year follow-up. Additionally, correlation analyses were executed to explore potential correlations between ucOC, cOC, and MoCA scores in both OP patients and healthy controls. Voxel-wise two-sample *T*-tests were performed to identify differences in grey matter volume (GMV) between OP patients and healthy controls, with age, and years of education as covariates. Multiple comparisons were conducted using the False Discovery Rate (FDR) method, setting a voxel-level *p*-value threshold at 0.001 and a cluster significance level at *p* < 0.05. The mean GMV within the identified brain regions was extracted and correlated with clinical metrics at baseline. To explore potential statistical causal relationships between altered GMV and clinical measures in OP patients, mediation analyses were performed. Given the absence of empirical evidence indicating the directionality of associations among ucOC, GMV, and MoCA scores, two models were considered: (1) GMV as the mediator, with ucOC as the independent variable and MoCA as the dependent variable; and (2) GMV as the mediator, with MoCA as the independent variable and ucOC as the dependent variable. The mediation effects were assessed using bootstrapped mediation analysis to evaluate the substantial difference between the total effect (path c) and the direct effect (path c’). The PROCESS macro (www.processmacro.org, version 2.16.3) in SPSS (IBM, version 23.0) was utilized, employing 5,000 bootstrap samples to establish 95% confidence intervals for the model components. Mediation was deemed statistically significant if the 95% confidence intervals did not include zero.

To eliminate any information leakage between feature determination and model development, the samples used for feature selection were entirely distinct from those used for model training and evaluation. Feature determination was performed exclusively in Dataset 1, the only cohort with serum ucOC measurements. Brain regions showing significant GMV differences between OP patients and healthy controls were screened for correlations with serum ucOC and MoCA scores, followed by bootstrap mediation analysis; regions exhibiting a significant mediation effect (the mPFC) were designated ucOC-associated neuroimaging markers, and binary masks of these regions were generated and fixed *a priori*. Model development was then performed exclusively in the independent Dataset 2 cohort: mean GMV within the pre-defined masks was extracted for each OP patient, and no feature screening or selection of any kind was performed in Dataset 2. Within Dataset 2, model performance was evaluated using leave-one-out cross-validation (LOOCV). In each of the 32 iterations, one patient was held out and the remaining 31 patients formed the training set. Five classifiers, logistic regression (LR), support vector machine (SVM), k-nearest neighbors (KNN), XGBoost, and CatBoost were trained using training samples and tested on the held-out subject. Because every patient was held out exactly once, the out-of-fold predictions from the 32 iterations were pooled to produce the final reported performance, representing the averaged prediction performance across all patients, and 95% confidence intervals (CIs) for the AUC were estimated from 2,000 bootstrap resamples of the pooled out-of-fold predictions. Patients with OP who experienced a decline of 4 points or more in MoCA scores at the 1-year follow-up were classified as being at risk for cognitive decline. This threshold was chosen *a priori* because a 4-point decline exceeds published minimal clinically important difference estimates for the MoCA and the reliable-change bounds derived from its test–retest reliability (28), thereby ensuring that positive cases reflect genuine clinical deterioration rather than measurement error.

## Results

### Demographic data

In the present study, two datasets were utilized. Dataset 1 serves as the feature discovery dataset (the only cohort with serum ucOC/cOC measurements), and Dataset 2 functions as an independent cohort for both neuroimaging (VBM) replication and machine learning model development using the fixed ucOC-associated imaging marker; serum ucOC and cOC were not measured in Dataset 2. It is important to note that no significant differences were found between the groups concerning age, years of education, or bone mineral density (BMD) (Table 1, *p* > 0.05).

**Table 1:** Demographic data and clinical assessment results of osteoporosis patients and healthy controls for both discovery dataset (Dataset 1) and testing dataset (Dataset 2).

Variables	Dataset 1			Dataset 2		
	OP	HC	*p* value	OP	HC	*p* value
Age	56.7 ± 7.6	57.2 ± 8.4	0.80	54.2 ± 6.6	54.4 ± 7.2	0.90
Years of education	7.2 ± 8.3	8.6 ± 7.6	0.91	7.4 ± 7.6	7.2 ± 8.1	0.91
BMD	−2.8 ± 1.1	0.7 ± 2.3	<0.001	−2.7 ± 1.4	0.7 ± 3.2	<0.001
MoCA (baseline)	20.7 ± 1.3	26.8 ± 1.3	<0.001	20.6 ± 2.5	NA	NA
MoCA (follow-up)	16.7 ± 1.2	NA	NA	19.2 ± 2.1	NA	NA
cOC	5.3 ± 0.6	7.1 ± 1.2	<0.001	NA	NA	NA
ucOC	2.2 ± 0.8	4.9 ± 0.9	<0.001	NA	NA	NA

BMD, Bone Mineral Density; MoCA, Montreal Cognitive Assessment; cOC, carboxylated osteocalcin; ucOC, undercarboxylated osteocalcin.

### Clinical assessments

In our discovery dataset, patients with OP demonstrated significantly lower MoCA scores compared to healthy controls, as illustrated in Figure 1a (left panel). This result indicates that individuals with OP exhibit diminished cognitive function. Furthermore, we observed a significant lower level of both serum undercarboxylated and carboxylated osteocalcin (ucOC, cOC) in OP patients when compared to healthy controls, as shown in the middle and right panels of Figure 1a. Notably, at the 1-year follow-up, a decline in cognitive performance was found in OP patients across both the discovery dataset (Figure 1b, left panel) and the testing dataset (Figure 1b, right panel).

**Figure 1:**
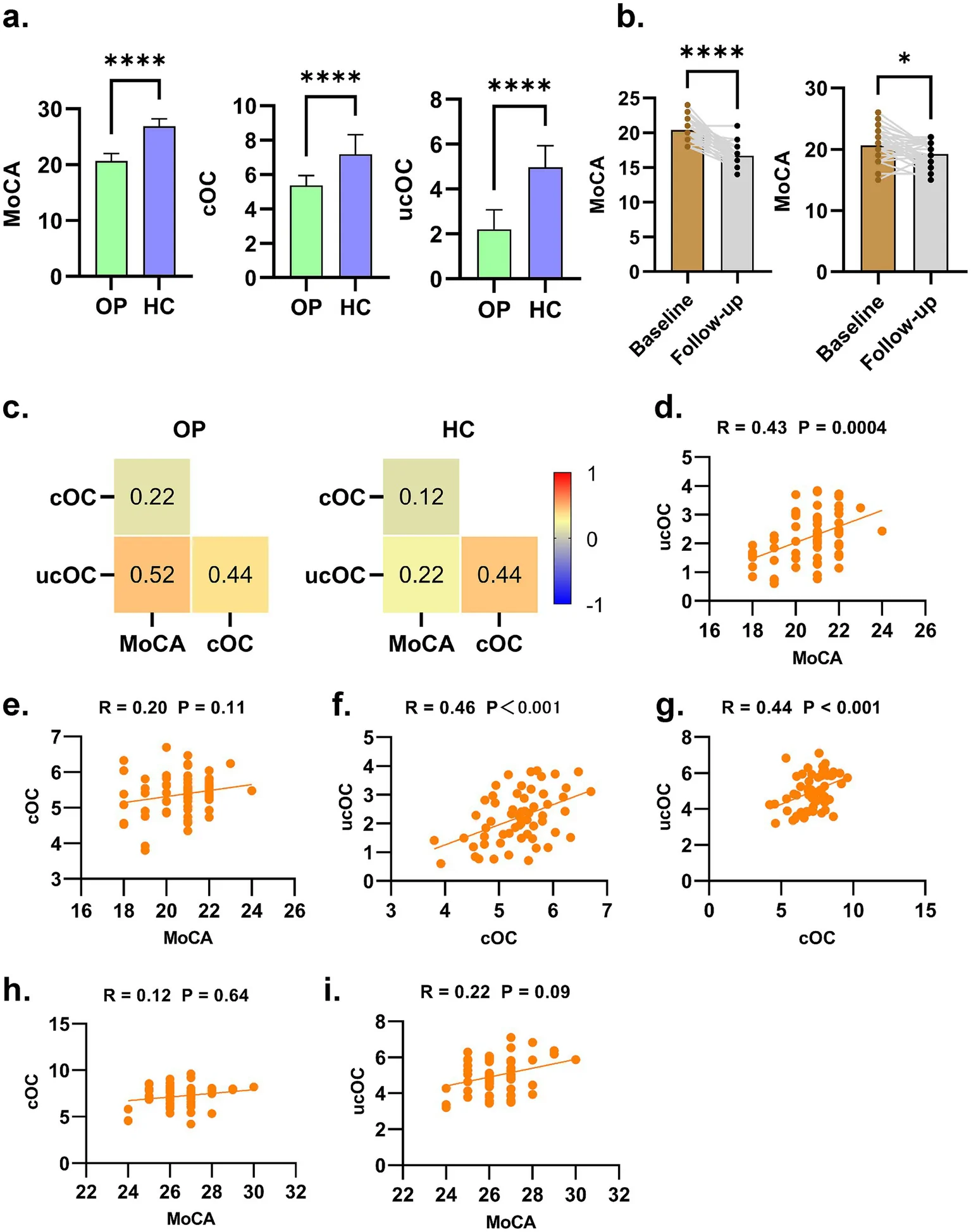
Clinical assessment of osteoporosis (OP) patients and healthy participants (HC). **(a)** Differences in carboxylated osteocalcin (cOC), Montreal Cognitive Assessment (MoCA) and undercarboxylated osteocalcin (ucOC) level between OP and HC in discovery dataset. **(b)** Longitudinal changes of MoCA scores in discovery dataset (left panel) and testing dataset (right panel). **(c)** Heatmaps for correlation coefficients among MoCA, cOC and ucOC in OP and HC group within discovery dataset. **(d–f)** Scatter plots for correlations among ucOC, MoCA and cOC in OP patients within discovery dataset. **(g–i)** Scatter plots for correlations among ucOC, MoCA and cOC in HC group within discovery dataset.

Additionally, a positive correlation was identified between serum levels of ucOC and MoCA scores in OP patients at baseline, as depicted in Figures 1c,d (*R* = 0.43, *p* < 0.001). In contrast, no significant correlation was found between cOC and MoCA scores in this patient group (Figure 1e). Moreover, a positive correlation was observed between serum levels of cOC and ucOC in OP patients (Figure 1f). It is noteworthy that a positive correlation between serum levels of cOC and ucOC was also present in the healthy control group (Figure 1g). However, among healthy controls, no significant associations were detected between ucOC and MoCA scores (Figure 1h) or between cOC and MoCA scores (Figure 1i).

### VBM analyses

OP patients exhibited significantly reduced gray matter volume (GMV) in the medial prefrontal cortex (mPFC), precuneus, and middle frontal cortex compared to healthy controls in the discovery dataset, alongside increased GMV in the calcarine gyrus (Figure 2a; Table 2). These findings were corroborated by the testing dataset, which also showed lower GMV in the mPFC and precuneus among OP patients (Figure 2b; Table 2). The violin plot in Figure 2c illustrates the GMV differences in the mPFC between OP patients and controls in the discovery dataset. Notably, in OP patients, mPFC GMV positively correlated with MoCA scores at baseline (Figure 2d; *R* = 0.47, *p* < 0.001). Furthermore, levels of undercarboxylated osteocalcin (ucOC) demonstrated a positive correlation with mPFC GMV (*R* = 0.48, *p* < 0.001, Figure 2e). In contrast, no correlation was observed between carboxylated osteocalcin (cOC) and mPFC GMV in OP patients (Figure 2f; *R* = 0.23, *p* = 0.06).

**Figure 2:**
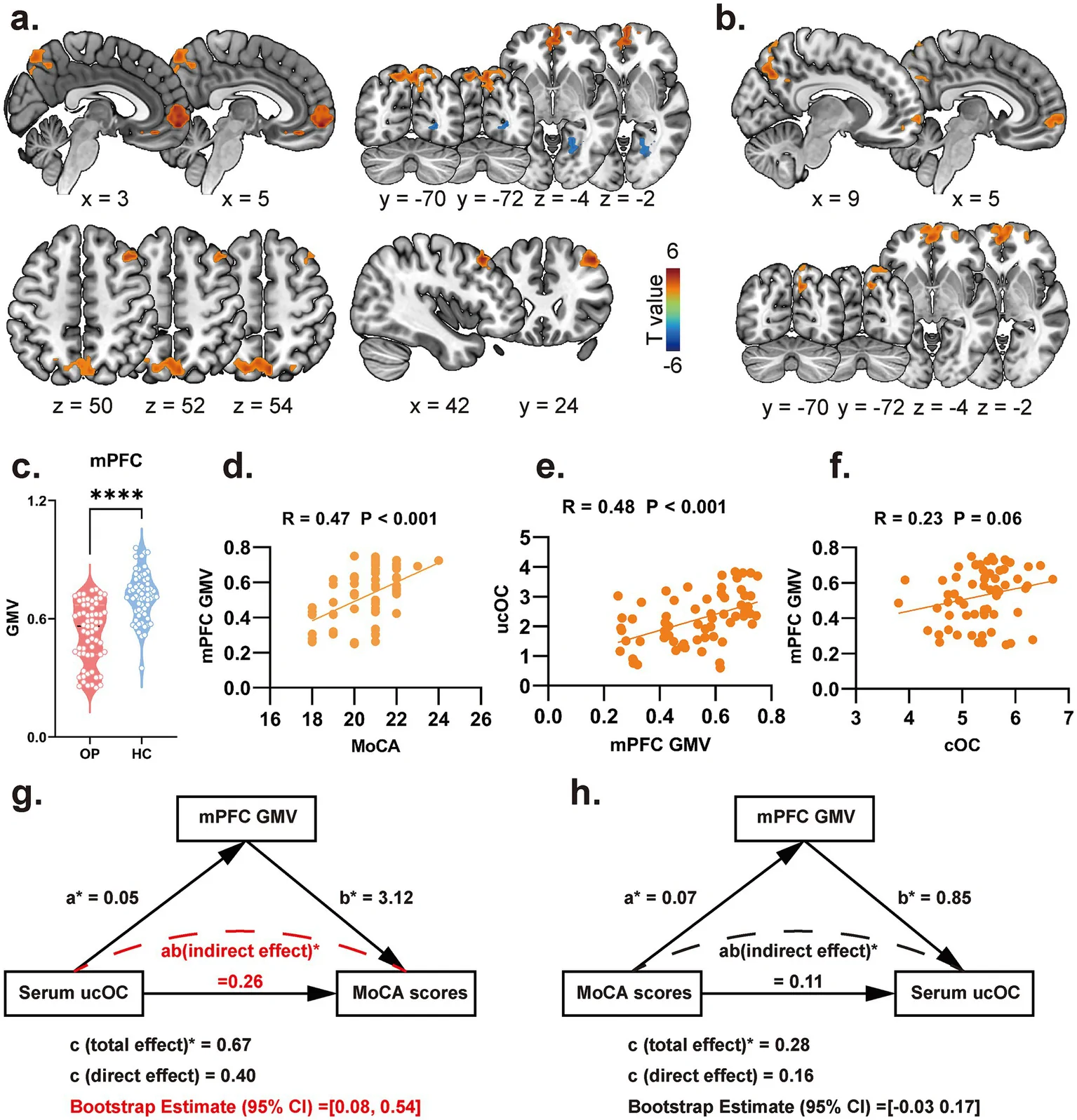
**(a)** Spatial distribution of grey matter volume (GMV) alterations within OP patients compared to healthy controls (HC) within discovery dataset (HC-OP). **(b)** Spatial distribution of GMV alterations within OP patients compared to HC in testing dataset (HC-OP). **(c)** GMV differences between OP and HC within medial prefrontal cortex (mPFC). **(d–f)** Scatter plots for correlations among mPFC GMV, carboxylated osteocalcin (cOC), Montreal Cognitive Assessment (MoCA) and undercarboxylated osteocalcin (ucOC) in OP patients within discovery dataset. **(g, h)** Mediation analyses identifying path association among ucOC, mPFC GMV and MoCA scores in OP patients within discovery dataset.

**Table 2:** Brain regions exhibited significant group differences between osteoporosis patients and healthy controls.

Brain region	Peak *T* value	Cluster size	Peak MNI Coordinates (*x*, *y*, *z*)		
Dataset 1 (Discovery dataset) (HC-OP)					
Left mPFC	6.51	1,480	3	60	−9
Bilateral precuneus	5.53	993	−3	−51	69
Left lingual gyrus	−4.1	426	−15	−54	−12
Right MFG	4.72	480	45	24	48
Dataset 2 (Testing dataset) (HC-OP)					
Left mPFC	4.43	863	−3	57	0
Right precuneus	4.20	632	9	−66	39

mPFC, medial prefrontal cortex; mPFC, medial prefrontal cortex; MFG, middle frontal gyrus.

To further investigate the associations between mPFC GMV and clinical metrics, we conducted mediation analyses. Within the discovery dataset, we identified specific interactions involving serum ucOC levels, mPFC GMV, and MoCA scores in OP patients. As depicted in Figure 2g, the direct effect of serum ucOC on MoCA scores was positive, quantified at 0.40, indicating that higher ucOC levels correlate with improved cognitive performance, as measured by MoCA. Additionally, an indirect positive effect was observed, suggesting that reduced mPFC GMV statistically mediates the relationship between ucOC and cognitive function. This indirect effect was calculated at 0.20, with a 95% bootstrap confidence interval ranging from 0.08 to 0.54, which excludes zero and thus confirms statistical significance.

Conversely, we explored an alternative model in which mPFC GMV served as the mediator, MoCA scores were the dependent variable, and ucOC was the independent variable. This analysis revealed a different pattern of relationships, indicating that the directionality of the statistical causal pathway significantly impacts the observed effects (Figure 2h). Collectively, these findings underscore the complex interplay among ucOC, mPFC GMV, and cognitive function in OP patients, highlighting the potential for bidirectional interactions and the critical importance of considering the direction of causality when interpreting the effects of these variables on cognitive performance.

### Machine learning prediction

The XGBoost (Figure 3a) model exhibited the highest AUC of 0.84, reflecting its superior discriminative power. The Support Vector Machine model also demonstrated strong performance with an AUC of 0.81 (Table 3). In contrast, the Logistic Regression model recorded the lowest AUC at 0.68, indicating it was the least effective among the tested models. The ROC curve for the XGBoost model is closest to the top-left corner, further reinforcing its position as the best-performing model. Conversely, the LR model’s curve is nearest to the diagonal line, which signifies a model with no discriminative ability (Figure 3b). Panel c presents the Decision Curve Analysis for each model, which evaluates the net benefit of employing the model’s predictions for decision-making across different threshold probabilities. The DCA results indicate that the XGBoost model offers the highest net benefit across a range of threshold probabilities, positioning it as the most valuable model for clinical decision support. The SVM and CatBoost models also demonstrate significant net benefits, while the LR and K-nearest neighbors models show less utility, particularly at lower threshold probabilities. Overall, these findings suggest that XGBoost and SVM are the most effective models for the classification task, with XGBoost emerging as the standout performer in terms of AUC, ROC, and DCA.

**Figure 3:**
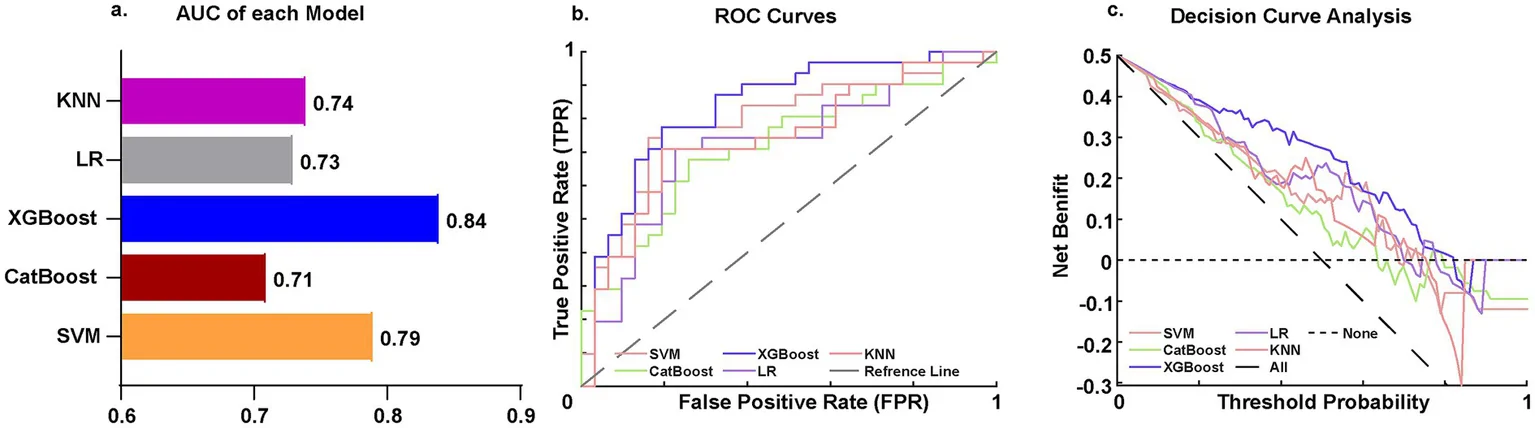
Machine learning analyses. **(a)** The area under curve (AUC) for machine learning models. **(b)** Receiver operating characteristic curves (ROC) for machine learning models. **(c)** Decision curve analysis for all machine learning models. KNN, K-Nearest Neighbors; LR, Logistic Regression; XGBoost, eXtreme Gradient Boosting; CatBoost, Categorical Boosting, SVM, Support Vector Machine.

**Table 3:** The model performances for machine learning models.

Model	AUC (95% CI)	Accuracy	Sensitivity	Specificity	Brier score
SVM	0.81 (0.78, 0.83)	73.53	0.76	0.71	0.18
CatBoost	0.73 (0.68, 0.76)	66.18	0.71	0.62	0.21
XGBoost	0.84 (0.79, 0.88)	82.35	0.82	0.82	0.17
LR	0.69 (0.63, 0.74)	61.76	0.76	0.47	0.23
KNN	0.77 (0.74, 0.80)	70.59	0.65	0.76	0.2

KNN, K-Nearest Neighbors; LR, Logistic Regression; XGBoost, eXtreme Gradient Boosting; CatBoost, Categorical Boosting; SVM, Support Vector Machine.

## Discussion

Over the past decade, the relationship between osteoporosis and cognitive decline has garnered substantial research attention, reflecting the growing recognition that these age-related conditions are mechanistically intertwined rather than merely coincident (29, 30). Numerous epidemiological and clinical studies have consistently demonstrated that individuals with osteoporosis face a substantially elevated risk of cognitive impairment, with recent meta-analyses reporting odds ratios exceeding 2.0 (31). However, the precise causal pathways linking skeletal deterioration to cognitive dysfunction remain incompletely understood, and current diagnostic strategies lack the sensitivity to identify high-risk patients before clinically overt cognitive decline manifests. In this context, our study makes several important contributions by elucidating the specific role of undercarboxylated osteocalcin (ucOC) as a mechanistically relevant biomarker, identifying medial prefrontal cortex (mPFC) grey matter volume as a neuroimaging correlate, and demonstrating the translational utility of integrating these multimodal measures through machine learning.

The brain-bone axis provides a compelling conceptual framework for understanding the OP-cognition nexus (7). Central to this axis is osteocalcin, a bone-derived hormone that exists in two principal forms: carboxylated osteocalcin (cOC), which is largely confined to the bone matrix, and undercarboxylated osteocalcin (ucOC), which is released into systemic circulation during osteoclastic resorption and represents the hormonally active form (32, 33). Our findings align with and extend prior evidence that ucOC, but not cOC, is the biologically relevant species for neuroprotection. Unlike cOC, ucOC can efficiently cross the blood–brain barrier and bind to neuronal Gpr158 receptors in regions critical for cognition, including the brainstem, hippocampus, and prefrontal cortex (34, 35). In rodent models, ucOC has been shown to prevent neuronal apoptosis, enhance synaptic plasticity, and modulate the synthesis of key neurotransmitters—including serotonin, dopamine, and GABA—thereby exerting broad neuroprotective effects (36, 37). Our observation that serum ucOC levels, but not cOC levels, positively correlate with both cognitive performance and mPFC GMV in OP patients strongly supports the biological specificity of ucOC in human brain health. Furthermore, the absence of significant ucOC-cognition associations in healthy controls suggests that the neuroprotective relevance of ucOC becomes particularly salient under pathological conditions, such as the low ucOC state characteristic of osteoporosis.

A particularly noteworthy finding of our study is the identification of the mPFC as a key structural substrate linking ucOC to cognitive function. The mPFC is integral to executive function, working memory, and emotional regulation—all domains that decline early in neurodegenerative disease (12, 38). Our mediation analysis provides robust statistical evidence that mPFC GMV significantly mediates the relationship between serum ucOC and MoCA scores, with an indirect effect of 0.20 and a 95% confidence interval excluding zero. This finding supports a mechanistic model in which reduced circulating ucOC in OP patients diminishes neurotrophic signaling to the mPFC, leading to progressive grey matter atrophy and consequent cognitive impairment. Importantly, both the discovery and testing datasets independently confirmed mPFC volume reduction in OP patients, underscoring the reproducibility and generalizability of this neuroimaging biomarker. From a translational perspective, mPFC GMV thus emerges as a quantifiable, non-invasive neuroimaging marker that captures the cerebral consequences of systemic ucOC deficiency.

Beyond elucidating mechanistic pathways, our study advances the field by demonstrating the clinical utility of an integrative multimodal prediction framework. Traditional univariate biomarkers, while informative, often exhibit insufficient discriminative power for individualized risk prediction due to the multifactorial nature of cognitive decline. By contrast, our machine learning approach synergistically combines serum ucOC—a peripheral biomarker of bone endocrine function—with mPFC GMV—a central neuroimaging marker of structural integrity—to achieve robust predictive performance. The XGBoost model attained an AUC of 0.84 (95% CI 0.79, 0.88) in the held-out test set, while Decision Curve Analysis confirmed its superior net benefit across clinically relevant threshold probabilities. It must be emphasized that this performance derives from internal validation within Dataset 1 only: serum ucOC and cOC were not measured in Dataset 2, which was collected primarily to replicate the structural neuroimaging findings on an independent scanner platform, and osteocalcin assays were not part of its acquisition protocol [authors to confirm the exact logistical reason]. Consequently, external validation of the complete multimodal model—requiring a prospective cohort in which serum ucOC/cOC assays, structural MRI, and longitudinal MoCA assessments are all obtained under harmonized protocols—remains an essential next step before clinical translation. This multimodal integration addresses several critical challenges in predictive medicine: it leverages complementary information across biological scales (molecular and macrostructural), accommodates non-linear interactions between predictors, and provides individualized probabilistic risk estimates that can inform clinical decision-making. For practicing clinicians, the ability to combine a simple blood test (ucOC) with structural MRI to accurately stratify OP patients according to their 1-year cognitive decline risk represents a significant step toward precision medicine in geriatric care.

The broader implications of our findings extend to the conceptualization of osteoporosis itself. Our results corroborate the view that osteoporosis is far more than a skeletal disorder—it is a systemic condition with profound implications for brain structure and function (32, 33). The positive correlation between serum ucOC and cognitive function observed in our OP cohort is consistent with prior reports in type 2 diabetes patients (39), further establishing ucOC as a transdiagnostic biomarker of cognitive resilience. Osteocalcin, functioning as an anti-aging protein, may hold therapeutic potential beyond bone health, with ucOC supplementation or bone-targeted interventions representing novel strategies to preserve cognitive function in aging populations (40). Moreover, the identification of mPFC atrophy as a structural mediator suggests that neuroimaging monitoring could serve as a surrogate endpoint in clinical trials of bone-targeted cognitive interventions.

### Limitation

Our current study has several limitations that necessitate careful consideration. Firstly, it is limited to the collection and analysis of brain structural data; functional aspects of the brain, including the Amplitude of Low-Frequency Fluctuations (ALFF) and Regional Homogeneity (ReHo), require additional investigation. Secondly, the study only examines the serum levels of osteocalcin (OCN); further research is required to explore other bone-derived proteins. Thirdly, while our focus has been on the medial prefrontal cortex (mPFC), future studies should aim to evaluate the predictive capacity of brain features through a data-driven approach. Fourthly, the mPFC is composed of various distinct subregions, and this study has not examined the functional and structural alterations in the mPFC subregions, thereby limiting our understanding of the detailed mechanisms involved. Fifthly, although we employed a two-stage leakage-free design with feature discovery in Dataset 1 and model evaluation in the independent Dataset 2, the prediction model was validated using only the ucOC-associated neuroimaging marker (mPFC GMV); the complete multimodal model directly incorporating serum ucOC measurements remains to be externally validated in a biomarker-complete cohort. The moderate sample size also limits the precision of the performance estimates, as reflected in the confidence intervals. Sixthly, the 4-point MoCA decline threshold, while justified by published studies and selected to avoid the class imbalance that would result from 3-point (too many positives) or 5-point (too few positives) cutoffs in our sample, was not subjected to sensitivity analysis across alternative thresholds; the robustness of model performance to cutoff variation warrants investigation in larger cohorts. Finally, residual confounding factors such as physical activity, dietary vitamin D intake, and socioeconomic status were not fully captured, despite our exclusion criteria and covariate adjustment for age and education.

## Conclusion

In conclusion, our study establishes undercarboxylated osteocalcin (ucOC) as a mechanistically relevant, circulating biomarker of cognitive resilience in osteoporosis, and identifies medial prefrontal cortex grey matter volume (mPFC GMV) as its structural neuroimaging correlate. Diminished serum ucOC in age-related osteoporosis compromises neuroprotective signaling, precipitating mPFC atrophy and cognitive decline. Importantly, machine learning algorithms yields a promising multimodal predictive framework that, under leakage-free internal validation, achieved good discriminative accuracy for 1-year cognitive decline risk; external validation in biomarker-complete cohorts is warranted.

## Data Availability

The raw data supporting the conclusions of this article will be made available by the authors, without undue reservation.
