# Supplementary material for: Integrative multimodal framework combining undercarboxylated osteocalcin and medial prefrontal cortex morphometry for prediction of cognitive decline in osteoporosis: a machine learning study
Source: Front Neurol. 2026 Sep 16;17:1950788. doi: 10.3389/fneur.2026.1950788 (PMC13623753; doi:10.3389/fneur.2026.1950788)
Supplement: Supplementary file 1 [file Supplementary_file_1.DOCX]

**Supplementary Methods**

**Patient Population**

The present study received approval from the ethics review committee of China-Japan Friendship Hospital, and informed written consent was obtained from each participant. We collected two datasets for this study. For Dataset 1, subjects who met the inclusion criteria were recruited from China-Japan Friendship Hospital by posting a recruitment notice, from July 2020 to August 2022. This group included sixty-three right-handed patients with osteoporosis (OP) and sixty right-handed healthy controls, matched for age and education. For Dataset 2, participants were recruited from China-Japan Friendship Hospital, from July 2022 to August 2023. This set consisted of thirty-two right-handed patients with OP and thirty-one right-handed healthy controls, also matched for education. To eliminate the potential influence of postmenopausal hormones on bone density, all participants in both datasets were male. This design helps ensure that any observed effects on bone density and cognitive function are not confounded by hormonal differences, providing clearer insights into the relationship between age related osteoporosis and cognitive decline.

The diagnostic criteria for osteoporosis (OP) were determined in accordance with the World Health Organization's guidelines [1, 2]. Specifically, inclusion required a bone mineral density (BMD) T-score of -2.5 or below, measured at either the femoral neck or the spine.

The criteria for exclusion comprised the following conditions: (1) being female; (2) presence of other comorbidities influencing bone metabolism, such as hypercortisolism, hyperthyroidism, glucocorticoid use, and connective tissue disorders; (3) a history of psychiatric or neurological disorders; (4) existence of additional complications, including diabetes or coronary artery disease; (5) abuse of alcohol or drugs; (6) use of hormonal medications; (7) contraindications for undergoing an MRI scan; (8) presence of brain abnormalities, such as tumors, cerebral infarctions, hemorrhages, or vascular malformations. Individuals exhibiting any of the aforementioned conditions were excluded from participating in this study.

**Clinical Assessment**

In both datasets, cognitive performance was assessed for patient cohorts and healthy controls during the initial data collection phase and then again after a one-year follow-up. The Montreal Cognitive Assessment (MoCA), a widely acknowledged instrument for detecting Mild Cognitive Impairment (MCI), was utilized, noted for its high sensitivity as reported by Hobson in 2015 [3]. A MoCA score of 26 or higher signifies normal cognitive function. To compensate for educational variations, individuals with less than 12 years of formal education received an additional point. The MoCA assessments were administered in a quiet setting by experienced examiners.

Additionally, for participants in dataset 1, blood samples were collected in the morning after a minimum of 8 hours of fasting. Serum was obtained by centrifugation at 3000 r/min for 10 minutes. Serum undercarboxylated osteocalcin (ucOC) levels were measured using the ELISA method (MK118, Takara Bio Inc, Japan), with intra-assay coefficients of variation ranging from 4.58% to 6.66%, and inter-assay coefficients of variation from 5.67% to 9.87%. Serum carboxylated osteocalcin (cOC) levels were also determined by ELISA (MK111, Takara Bio Inc, Japan), with intra-assay coefficients of variation between 3.3% and 4.8%, and inter-assay coefficients of variation between 0.7% and 2.4%.

**Data Acquisition and preprocessing**

Dataset 1: High-resolution T1-weighted structural images were acquired. Brain images were obtained using a Siemens 3T Trio MRI scanner with a Magnetization Prepared Rapid Gradient-echo (MP-RAGE) sequence. The imaging parameters were as follows: inversion time of 900 milliseconds, repetition time/echo time of 1900/2.52 ms, field of view of 256 × 256 mm, flip angle of 9 degrees, and a voxel resolution of 1 × 1 × 1 m^3^. A total of 176 slices were acquired at a slice thickness of 1 mm.

Dataset 2: Structural imaging data were obtained using a 3.0-T magnetic resonance imaging (MRI) scanner (Discovery MR750, General Electric). A three-dimensional T1-weighted imaging sequence (3DT1WI) was employed, with the following parameters: sagittal acquisition mode, repetition time (TR) of 7.8 milliseconds, echo time (TE) of 3.0 milliseconds, inversion time (TI) of 450 milliseconds, flip angle of 13 degrees, field of view of 256 × 256 mm, matrix size of 256 × 256, 180 slices, and a slice thickness of 1.0 mm.

Participants were instructed to maintain immobility to ensure the highest possible image resolution.

**Voxel-Based Morphometry (VBM)**

Structural MRI data were processed using the Computational Anatomy Toolbox (CAT) within the Statistical Parametric Mapping software suite (SPM12, version 6906; Wellcome Trust Centre for Neuroimaging), which was interfaced through MATLAB 2023a (MathWorks Inc., Natick, Massachusetts, USA). Initial quality control steps involved the meticulous inspection of images to exclude any with imaging artifacts or significant anatomical variations. Subsequently, manual refinements were applied to align the images with the anterior commissure, thereby refining the registration process. The images underwent a meticulous segmentation protocol, which was designed to discern white matter, grey matter, and cerebrospinal fluid with enhanced precision and reliability. Post-segmentation, the images were resampled to isotropic voxels of 1.5 mm and registered using the Diffeomorphic Anatomical Registration Through Exponentiated Lie Algebra (DARTEL) template creation toolkit to enhance inter-subject correspondence. Standardization to the Montreal Neurological Institute (MNI) template space ensued, along with modulation based on the Jacobian determinant to account for spatial normalization adjustments. The modulated images were then smoothed with an isotropic Gaussian kernel with a Full-Width at Half-Maximum (FWHM) of 8 mm to mitigate artifacts and signal noise resulting from image standardization and head motion. The smoothed images, representing grey matter volume (GMV), were subjected to z-scoring for further analyses. It is noteworthy that the FWHM of the Gaussian kernel, commonly used in voxel-based morphometry (VBM) analyses, typically ranges from 8 to 12 mm, as supported by previous research. Shen et al.'s study underscored the advantage of smaller kernels in VBM analyses, with optimal performance varying with group size [4]. The study's findings suggested that an 8-10 mm kernel provides the highest accuracy for atrophy detection in studies with smaller sample sizes. In contrast, a 6 mm kernel is more effective for larger datasets with over 50 participants. Considering the modest sample size in our research, we opted for an 8 mm FWHM isotropic Gaussian kernel to enhance the precision of atrophy detection. This choice balances the need for accurate detection while accounting for the specific characteristics of our dataset.

**Supplementary References**

1.	Ivanova, S., et al., *Osteoporosis: Therapeutic Options.* Folia Med (Plovdiv), 2015. **57**(3-4): p. 181-90.

2.	Wáng, Y.X.J., et al., *Revision of the 1994 World Health Organization T-score definition of osteoporosis for use in older East Asian women and men to reconcile it with their lifetime risk of fragility fracture.* Skeletal Radiol, 2024. **53**(4): p. 609-625.

3.	Jia, X., et al., *A comparison of the Mini-Mental State Examination (MMSE) with the Montreal Cognitive Assessment (MoCA) for mild cognitive impairment screening in Chinese middle-aged and older population: a cross-sectional study.* BMC Psychiatry, 2021. **21**(1): p. 485.

4.	Shen, S. and A. Sterr, *Is DARTEL-based voxel-based morphometry affected by width of smoothing kernel and group size? A study using simulated atrophy.* J Magn Reson Imaging, 2013. **37**(6): p. 1468-75.
